# Patients with advanced oral squamous cell carcinoma have high levels of soluble E-cadherin in the saliva

**DOI:** 10.4317/medoral.21907

**Published:** 2017-10-21

**Authors:** Sandra López-Verdín, Juan-José Soto-Avila, Ana-Lourdes Zamora-Perez, Blanca-Patricia Lazalde-Ramos, Margarita de la Luz Martínez-Fierro, Rogelio González-González, Nelly Molina-Frechero, Mario-Alberto Isiordia-Espinoza, Ronell Bologna-Molina

**Affiliations:** 1 Health Sciences University Center,Instituto de Investigación en Odontología, Universidad de Guadalajara, Jalisco, México; 2 Head and Neck Department, Instituto Jalisciense de Cancerología, Jalisco, México; 3 Academic Unit of Human Medicine and Health, Universidad Autónoma de Zacatecas, Zacatecas, México; 4 Department of Research, School of Dentistry, Universidad Juárez del Estado de Durango, Durango, México; 5 Health Care Department, Universidad Autónoma Metropolitana, Mexico City, Mexico; 6 Farmacology Department, Faculty of Medicine, Universidad Autónoma de San Luis Potosí, San Luis Potosí, México; 7 Molecular Pathology area, School of Dentistry, Universidad de la Republica, Montevideo, Uruguay

## Abstract

**Background:**

The objective of this study was to assess the potential clinical value of the concentration of soluble salivary E-cadherin (sE-cadherin) compared with the clinical value of the presence of membranous E-cadherin (mE-cadherin) in oral squamous cell carcinoma tumor tissues.

**Material and Methods:**

Data regarding patient demographics, clinical stage, saliva and tumor tissue samples were collected. The saliva was analyzed for sE-cadherin protein levels and was compared to the mE-cadherin immunohistochemical expression levels in tumor tissues, which were assessed via the HercepTest® method. Patients without cancer were included in the study as a control group for comparisons of the sE-cadherin levels.

**Results:**

sE-cadherin levels in the saliva of patients without cancer were lower than those in patients with cancer, and the difference was statistically significant (*p*=0.031). Low mE-cadherin expression was statistically significantly associated with lymph node positivity (*p*=0.015) and advanced clinical stage (*p*=0.001). The inverse relationship between mE-cadherin and sE-cadherin was significant in terms of lymph node positivity (*p*=0.014) and advanced clinical stage (*p*=0.037).

**Conclusions:**

The results suggest that sE-cadherin levels are significantly increased in patients with oral cancer and that its low expression within the membrane as well as the progression of the disease appear to be inversely associated with levels of sE-cadherin in the saliva.

** Key words:**E-cadher.in, saliva, oral cancer.

## Introduction

Oral epithelial cells possess unique morphological characteristics, including extensive inter-cellular junctional complexes and stable cell-cell and cell-matrix adhesions (1). The decrease in the levels of cell-cell adhesion molecules is central to the development of aggressive carcinomas because the absence of these molecules enables the cancer cell to break free from the primary tumor and migrate to local and distant sites within the body to form new tumors (2).

E-cadherin belongs to the cadherin family of proteins, which was first described in connection with tissue specificity since these proteins were found in epithelial tissues (3). E-cadherin is a 120-kDa transmembrane glycoprotein encoded by the CDH1 gene located on chromosome 16q22.1a. It enables junctional adherence via the binding of the extracellular domains of E-cadherin molecules between adjacent cells and the binding of intra-cytoplasmic domains with catenins, which are cytoplasmic proteins that connect E-cadherin to the cytoskeleton (4,5). This allows the participation of E-cadherin in the transduction of signals that control several cellular events, including polarity, differentiation, growth and cell migration (6).

Although reduced expression of E-cadherin was once considered necessary for metastatic dissemination, several reports have documented invasive and aggressive tumors that nonetheless maintain E-cadherin expression (7-10).

It is now known that not only does its reduced expression play a role in the transition to a malignant phenotype and in cancer progression but that the proteolysis of E-cadherin by several proteases converts the mature 120-kDa E-cadherin to an extracellular N-terminal 80-kDa fragment termed soluble E-cadherin (sE-cadherin), which serves as a paracrine/autocrine signaling molecule in cancer (11).

It has been reported that the serum levels of sE-cadherin are higher in oncological patients than in healthy subjects (12-14). It is possible that similar results could be observed in saliva, which is a popular fluid for use in oral squamous cell carcinoma (OSCC) (1) research because saliva is present in a certain composition and responds to events in the oral cavity (15).

The goal of our research was to determine, for the first time, the concentration of salivary sE-cadherin in OSCC, to assess the membrane expression of E-cadherin, to correlate this expression with disease characteristics and to determine the association between sE-cadherin and membranous E-cadherin (mE-cadherin).

## Material and Methods

-Patients 

Individuals diagnosed with OSCC who were patients at the Jalisco Institute of Oncology between January 2013 and February 2015 were enrolled in the study. In all, 26 patients were enrolled (12 male and 14 female subjects, aged 61.9 ± 16.7 years). The reason for the greater number of female patients was simply because they volunteered in greater numbers.

In addition, samples from 10 individuals without OSCC who visited the clinic for teeth extraction due to orthodontic or prosthetic indications were included (4 male and 6 female patients, aged 57.3 ± 8.5 years) and were treated at the Civil Hospital “Dr. Juan I Menchaca”.

The stage of the disease was evaluated in the Surgical Head and Neck Oncology Department, and each tumor was analyzed clinically and by computerized tomography. The disease stages were then determined according to the TNM system (16).

Due to the finding that the diameter of the tumor and the invasion into lymphatic nodes are regarded as important clinical markers involved in prognosis, the lymphatic nodes were considered positive if patients presented metastasis in one or more lymphatic nodes. T stage and total TNM scores were redistributed in accordance with the NCI guidelines (17), and each case was determined to be early- or advanced-stage disease, as shown in  Table 1. According to this system, advanced T stage refers to tumors that are larger than 4 cm, whereas advanced TNM stage also incorporates tumors of a smaller size that are accompanied by positive lymphatic nodes with or without distant metastasis.

**Table 1:**
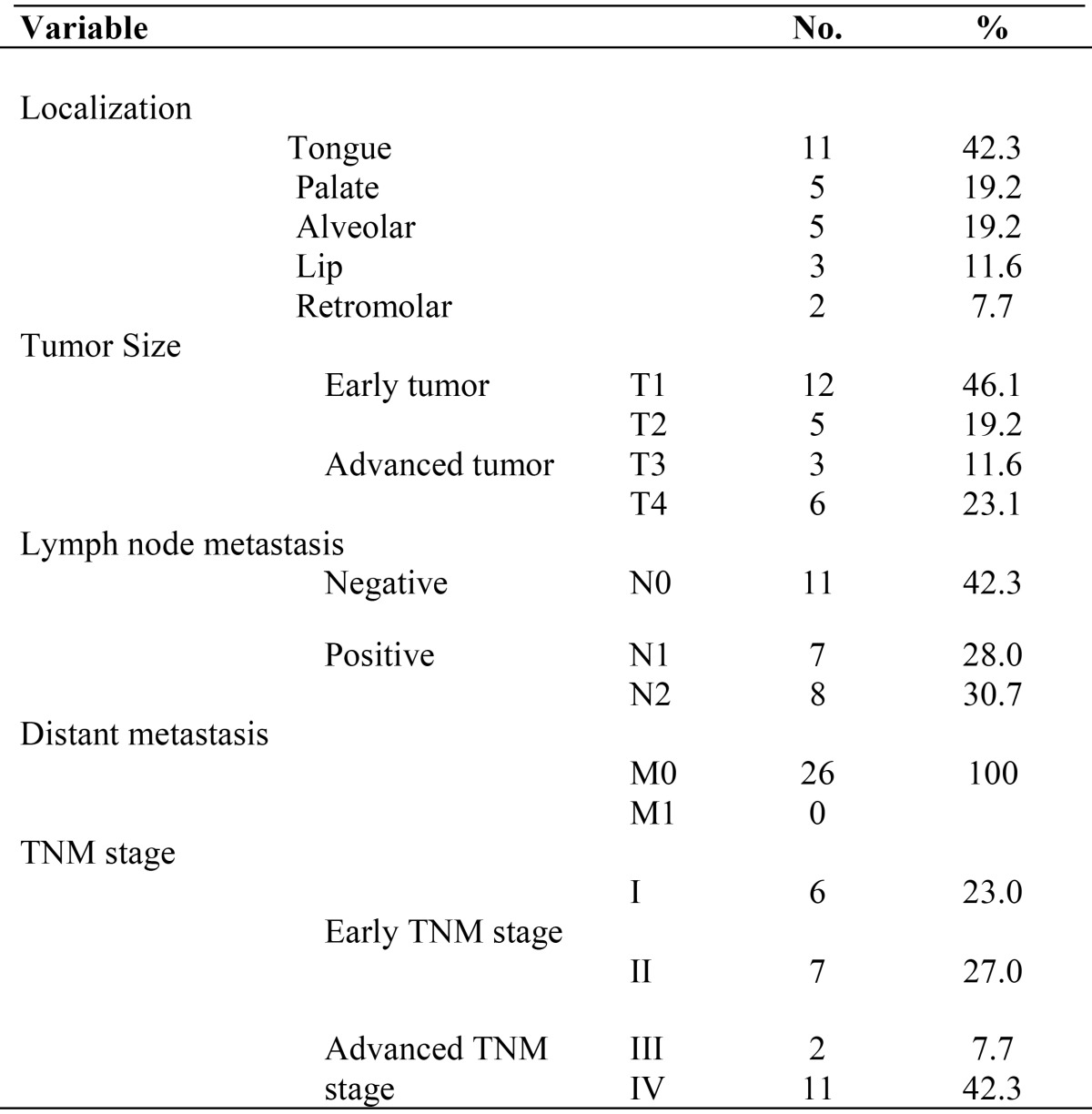
OSCC, oral squamous cell carcinoma; the T and clinical stages were redistributed in accordance with the guidelines of the NCI.

Confirmation of the diagnosis and the tumor grade (differentiation) was determined according to the WHO criteria (18). Both procedures were performed by an oral pathologist. Informed consent from each patient was included in the study protocol, which was submitted to and approved by the ethics committee of the institute. The authors have read the Declaration of Helsinki and have followed its guidelines in this investigation.

-Selection Criteria

Patients diagnosed with OSCC, who did not receive chemotherapy or radiotherapy, or any other oncological treatment including surgery, were enrolled in this study. Individuals without OSCC but who experienced epithelial autoimmune or inflammatory disease, those who presented isolated ulcerous disorders, e.g., traumatic disorders, or any other kind of lesion in the oral mucosa, and those with any oncological history were excluded from the study.

-Samples

Unstimulated saliva was collected between 9 and 11 AM before any surgical procedure was performed and was preserved according to a previously described protocol (19). Saliva was not collected from patients who had consumed alcohol, smoked tobacco or performed oral hygiene procedures up to 30 minutes before sample collection or from those who presented bleeding of the gums or of any other location within the oral cavity. Biopsy tissue samples were collected by an oncological surgeon and were divided into two groups, as follows: one to be sent to the hospital for diagnosis, and the other (measuring 5 mm to 1 cm) to be included in experimental procedures. The tissue was immediately fixed in 10% formaldehyde for a minimum of 24 hours and no longer than 48 hours and was later paraffin-embedded in the pathology laboratory of the Microbiology and Pathology Department; paraffin blocks were then sectioned for hematoxylin and eosin staining to confirm the diagnosis.

-Enzyme-linked Immunosorbent Assay (ELISA)

The concentration of sE-cadherin was measured in the saliva of 24 OSCC patients. Saliva samples from two patients could not be collected or were insufficient for detection. It was also difficult to obtain adequate saliva for the laboratory procedures. This was because patients with tumors in the oral cavity generally experience limited mobility or paralysis or due to the size of the tumor and because the saliva usually contains blood from tumor ulceration or is heavily contaminated because of oral hygiene (both are exclusion criteria according to the protocol).

We used the commercially available Quantikine® ELISA kit (R&D Systems, Minneapolis, MN, USA) according to the manufacturer’s instructions. Briefly, the saliva sample was diluted, pipetted into the wells of the titer plates and incubated with detection antibodies at room temperature for 1 to 2 hours. After incubation, the solution was aspirated, and the wells were washed 4 times. The substrate was added to each well for 20 minutes at room temperature followed by the addition of stop solution. Optical density was measured in an ELISA reader (Bio-hazard model Powean WH4101). All tests were performed in triplicate, and the concentration was calculated from a validated standard curve using a simple linear regression model. Data regarding the total concentration of proteins were unnecessary since the kit we utilized did not require this value; this kit is specifically designed for the quantification of human sE-cadherin and for the determination of its concentration in cell culture supernatants, serum, plasma, urine, and saliva, and therefore, the data are expressed as nanograms (ng) of E-cadherin per ml of saliva.

-Immunohistochemistry (IH)

Tissue samples from 26 cases were processed and evaluated. From the tissue blocks, 3-μm-thick sections were cut, placed on poly-L-lysine-coated slides, deparaffinized in a 60°C oven for 30 minutes and incubated in xylol for 5 minutes. The sections were then rehydrated in decreasing alcohol concentrations (absolute, 90, 70, and 50%) and washed in distilled water. To retrieve the epitopes, the tissue sections were heated in 10 mM sodium citrate solution at pH 9 in a microwave oven at a maximum power of 750 W for two cycles of 5 minutes each. The sections were then cooled to room temperature and washed with distilled water. Endogenous peroxidase was blocked with 0.9% hydrogen peroxide, and the samples were again washed with distilled water and phosphate-buffered saline solution (PBS, pH 7.4). The sections were incubated for 30 minutes with a primary antibody against E-cadherin (Clone NCH-38, 1:50, Dako Corp., Carpinteria, CA, USA); following this, the sections were incubated with a biotiny-lated anti-mouse/anti-rabbit secondary antibody and streptavidin/peroxidase complex (LSA-B+ labeled streptavidin-biotin, Dako Corp.) for 30 minutes each. The reaction products were then detected with 3,3′-diaminobenzidine-H2O2 (Dako Corp.).

Negative and positive controls were obtained from paraffin blocks containing samples of healthy mucosa and fibroma that were collected using a 5-mm punch and placed on the slides along with the samples. The slides were analyzed by an oral pathologist, and the cells were counted as previously described for the HercepTest® (DAKO, Carpinteria, CA, USA) for breast cancer (20). In this test, the staining intensity on the membrane and the percentage of stained cells are scored from 0 to 3+ as follows: 0, negative: no staining is observed or membrane staining is observed in <10% of the tumor cells; 1+, negative: faint/barely perceptible membrane staining is detected in >10% of tumor cells; 2+, weakly positive: weak to moderate complete membrane staining is visualized in >10% of tumor cells; and 3+, positive: strong and complete membrane staining is visualized in >10% of tumor cells. For statistical analyses, the scores were stratified into low (0 and 1+) and high (2+ and 3+) expression groups. The term mE-cadherin was created by associating this protein’s form of expression to its corresponding membrane region based on the presence of E-cadherin.

-Statistical Analysis

The sample size of cases included in this study was in agreement with a previous study in which sE-Cadherin was analyzed in head and neck cancer (21).

The size of the control groups was established by the hospital’s internal policy and was adopted for successful protocol authorization.

Data were captured and analyzed using descriptive and inferential non-parametric statistics (SPSS version 20). The sE-cadherin protein concentrations are expressed as median values because the Mann-Whitney U and Kruskal-Wallis tests for quantitative variables are commonly regarded as tests with which to determine medians within the population. Fisher’s Exact Test was used for qualitative variables because the number of samples for some of the analyses was less than or equal to 5. A *p* value <0.05 was considered statistically significant with a confidence interval (C.I.) of 95%.

## Results

In all, 26 patients with OSCC were included in this study. The TNM and clinical stages of the patients are shown in  Table 1.

The area where OSCC was most frequently found was the tongue (42.3%), followed by the alveolar mucosa (19.2%) and palate (19.2%). T1 and N0 were the stages most frequently reported among patients (46.1% and 42.3%, respectively), but a high percentage of patients in advanced clinical stages (42.3%) was also detected. None of the patients were determined to have distant metastasis (M0).

The histopathological assessment of tumor tissues established that 21 tumors (80.8%) were considered well-differentiated, 4 (15.4%) were considered moderately differentiated, and finally, only one case (3.8%) was considered poorly differentiated.

-sE-cadherin levels 

**Figure 1:**
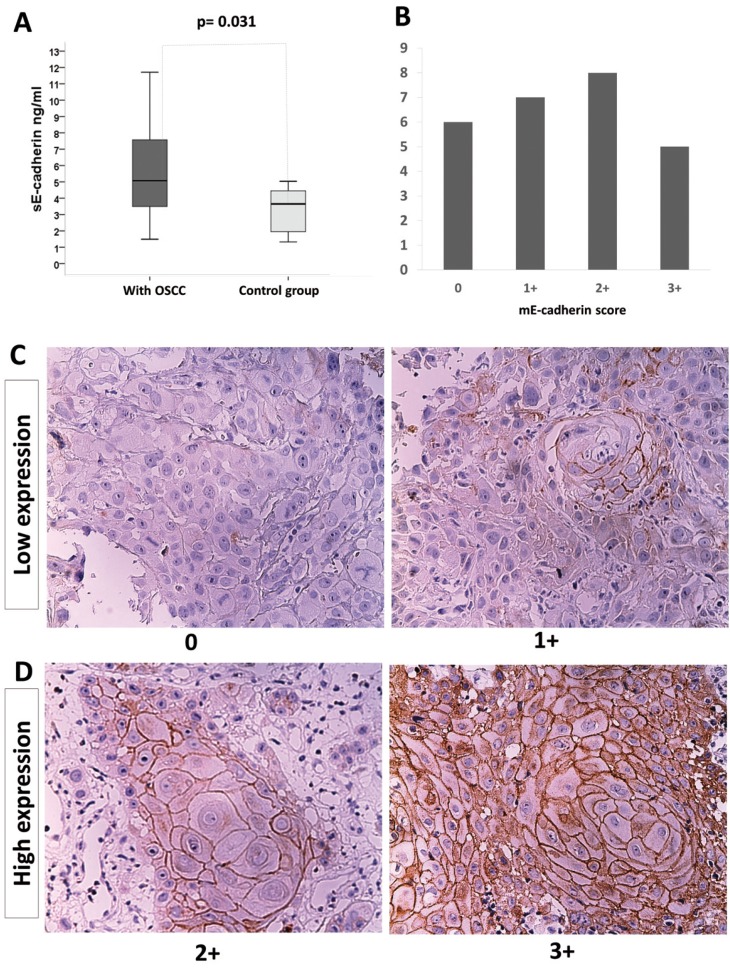
Levels of sE-cadherin as evaluated by ELISA, and the presence of mE-cadherin as determined by immunohistochemistry in OSCC. A) Concentration of sE-cadherin in the saliva from OSCC patients and controls (*p=0.031). Box: lower line - quartile Q1 (25%-quartile); middle line - median; and upper line - quartile Q3 (75%-quartile) (Wilcoxon-Mann-Whitney test, 95% C.I.). B) The expression of mE-cadherin in the tumor tissue was not predominantly high or low. The x-axis shows the score based on the HercepTest®. C) Low expression: 0, poorly differentiated carcinoma with absent mE-cadherin immunoreactivity (original magnification 20x); and 1+, well-differentiated carcinoma with poor or absent mE-cadherin expression (original magnification 20x). D) High expression: 2+, well-differentiated carcinoma with moderate and intense areas of mE-cadherin immunostaining (original magnification 20x); and 3+, well-differentiated carcinoma with evident, intense mE-cadherin immunostaining (original magnification 20x).

The median sE-cadherin level in the saliva of patients without cancer (median 3.64 ng/ml, minimum 1.32 ng/ml and maximum 5.03 ng/ml) was lower than that in the saliva of cancer patients (median 5.07 ng/ml, minimum 1.48 ng/ml and maximum 13.91 ng/ml), and the difference was statistically significant (*p*=0.031) (Fig. 1A). The range in the levels of sE-cadherin was broad. However, we must note that the maximum levels of sE-cadherin in controls did not exceed the median among the OSCC samples.

The difference in the levels of sE-cadherin was not statistically significant among cases that varied with respect to T stage (*p*=0.159), N stage (*p*=0.120), clinical stage (*p*=0.857), or tumor grade (*p*=0.273). We also did not observe a marked tendency toward any particular stage. M stage was not considered in this analysis since we did not evaluate a comparative group.

-mE-cadherin expression 

The frequency of mE-cadherin expression in tumor tissues according to the HercepTest® was as follows: 23.0%=0, 27.0%=1+, 30.7%=+2, and 19.2%=+3 (Fig. 1B).

A representative microscopic image of each HercepTest® score in stratified OSCC samples is shown in Figure 1C and D.

The presence of mE-cadherin was classified as either low or high based on the HercepTest®. No significant differences were found in mE-cadherin expression in tumors of different sizes (*p*=0.097); in contrast, statistically significant differences were observed between positive and negative lymph nodes (*p*=0.015) as well as between early and advanced clinical stages (*p*=0.001) ( Table 2).

**Table 2:**
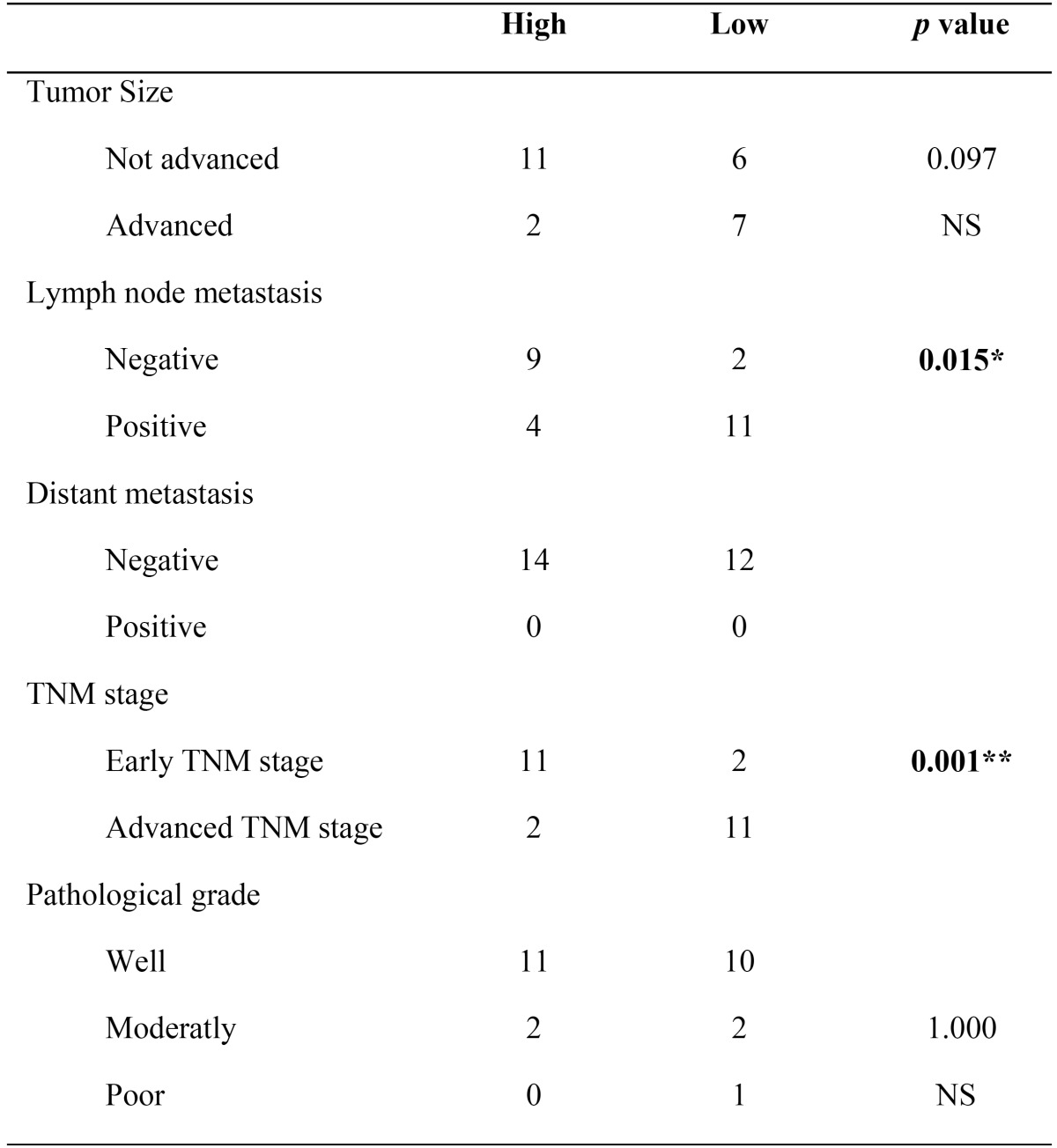
Fisher’s Exact Test with 95% C.I. NS: no significance; *p<0.05; and ** *p*≤0.001.

The degree of differentiation was not statistically significant (*p*=1.000) based on the presence of mE-cadherin.

-Expression of sE-cadherin and mE-cadherin

**Figure 2:**
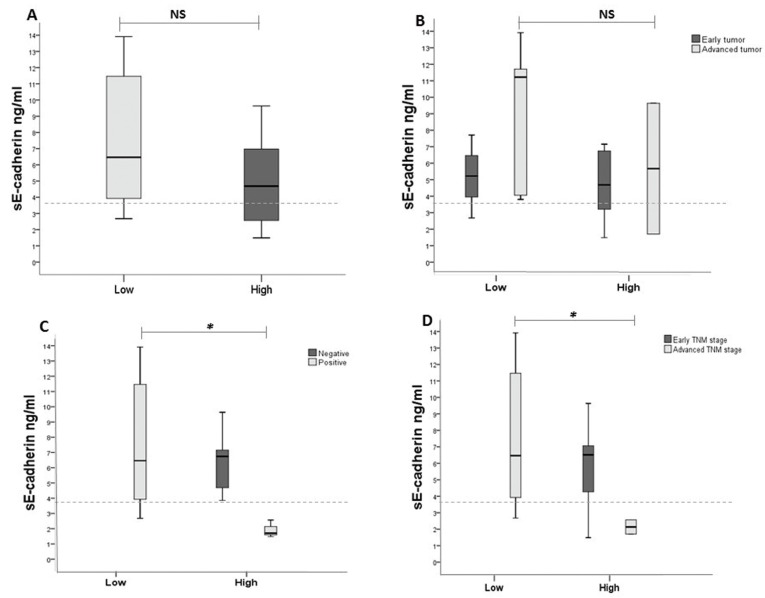
The levels of sE-cadherin as evaluated by ELISA and the expression of mE-cadherin as measured by immunohistochemistry were correlated with disease progression. A) Trend toward an inverse relationship: low expression of E-cadherin was associated with a high level of sE-cadherin, whereas high levels of E-cadherin were associated with low levels of sE-cadherin (*p*=0.178). B, C, D) This tendency was maintained in only the most advanced stages of the three clinical criteria. Advanced T stage cases showed an increase in the levels of sE-cadherin with low expression of mE-cadherin (*p*=0.245). In positive nodes, the levels of sE-cadherin were greater when mE-cadherin was expressed at low levels compared with the levels of sE-cadherin when mE-cadherin was expressed at high levels (*p=0.014). In cases at an advanced clinical stage, the levels of sE-cadherin when the expression level of mE-cadherin was low were greater than the levels of sE-cadherin when the expression level of mE-cadherin was high (*p=0.037). Box: lower line - quartile Q1 (25%-quartile); middle line - median; and upper line - quartile Q3 (75%-quartile) (Wilcoxon-Mann-Whitney test, 95% C.I.). Dotted line: median of sE-cadherin within the healthy control group.

A comparison of the levels of sE-cadherin with the presence of mE-cadherin did not reveal statistically significant differences. However, a trend toward an inverse relationship was observed: low levels of mE-cadherin were associated with high levels of sE-cadherin (median 6.46 ng/ml, minimum 2.67 ng/ml and maximum 13.91 ng/ml), whereas high levels of mE-cadherin were associated with low levels of sE-cadherin (median 4.68 ng/ml, minimum 1.48 ng/ml and maximum 9.63 ng/ml) (Fig. 2A).

This tendency was observed only in the most advanced stages. Advanced T stage cases showed an increase in the levels of sE-cadherin when the levels of mE-cadherin were low (median of 11.75, minimum of 3.75 and maximum of 14.16) compared with the levels of sE-cadherin when the levels of mE-cadherin were high (median of 6.32, minimum of 1.87 and maximum of 9.21), but this difference was not statistically significant (Fig. 2B).

Nevertheless, positivity in regard to the invasion of lymphatic nodes and advanced clinical stage did show statistically significant differences. The levels of sE-cadherin were higher when mE-cadherin was expressed at low levels (median of 6.63, minimum of 2.69 and maximum of 14.16) in positive nodes compared with the levels of sE-cadherin when mE-cadherin was robustly expressed (median of 1.83, minimum of 1.76 and maximum of 2.49) (*p*=0.014) (Fig. 2C). Similarly, the levels of sE-cadherin when mE-cadherin expression was low (median of 6.63, minimum of 2.69 and maximum of 14.16) in advanced clinical stages were greater than the levels of sE-cadherin in the presence of high mE-cadherin expression (median of 1.83, minimum of 1.76 and maximum of 2.49) (*p*=0.037) (Fig. 2D).

## Discussion

In this study, we found statistically significant differences in the saliva sE-cadherin levels between the OSCC group and the group without OSCC. The results of this study are consistent with those of Al Kassam *et al.*, who studied patients with head and neck cancer (21). That study showed statistically significant differences in the levels of sE-cadherin between the blood plasma of healthy individuals and that of cancer patients but not in terms of the TNM and clinical stages in a similar sample size. They also did not include comparisons regarding the tumor grade (differentiation).

An important difference between our study and other studies is the use of salivary fluid instead of blood plasma.

Despite the disadvantages we described concerning the methodology, we agree on the advantages in using saliva: collection of saliva is a non-invasive procedure, sample collectors do not need any special training, and the presence of normal materials (cells, DNA, RNA and proteins) and inhibitory substances is low (22). The detection of high levels of sE-cadherin in patients with oral cancer enables us to suggest the use of saliva as a tool for analysis when E-cadherin dysregulation needs to be determined. However, further studies are required to establish the features of sE-cadherin in the saliva and the relationship of sE-cadherin with the disease.

Since the degradation of the extracellular portion alone cannot lead to the total loss of E-cadherin protein, we also evaluated the expression of mE-cadherin (23).

The mE-cadherin expression results are consistent with those in many publications in which low expression is statistically significantly correlated with node invasion and advanced TNM stage in OSCC (24,25). However, various studies have not found a statistically significant association between low or high membranous expression and the size of the tumor, which is in agreement with our results (7-9,26,27).

The main obstacle against the use of mE-cadherin expression as a trustworthy marker is the occurrence of false-positive detection, in which aggressive, invasive tumors express mE-cadherin (7-10). It has been suggested that this is because many antibodies against E-cadherin may only target the extracellular or intracellular portions, and therefore, even with the loss of one portion of the protein, a positive immunoreaction may still be observed, which would affect the detection of malignancy.

These last observations also served to address the last objective of our study, which was to demonstrate new possible associations in OSCC via a comparison of the results of the immunohistochemical analysis to the levels of sE-cadherin by ELISA to interpret the extracellular events that accompany high or low mE-cadherin expression. The inverse relationship we observed was not statistically significant; however, when these values were grouped in accordance with node invasion and advanced TNM stage, this tendency became statistically significant. These results may be associated with the proteolytic events that occur in the tumor microenvironment. These proteolytic events enable neoplastic cells to migrate and invade other sites.

In contrast, a small number of OSCC cases exhibited high expression of mE-cadherin and higher median values of sE-cadherin than the control group. This finding allows for the possibility to distinguish tumors by revealing intracellular processes that mediate malignant pathways, independent of extracellular mE-cadherin proteolysis.

In addition, we need to consider the difference between statistical and biological significance, since other types of analyses were not included, e.g., gene expression analysis and/or expression analysis in the cytoplasmic region as well as the measurement of proteolytic enzymes that target E-cadherin. However, if our results are both statistically and biologically significant, a therapeutic strategy could be designed to target extracellular or intracellular proteolytic events.
